# The influence of OSPE and PBL on competency-based pharmacy student self-assessment

**DOI:** 10.1186/s12909-022-03246-5

**Published:** 2022-03-18

**Authors:** Justyna Dymek, Tomasz Mateusz Kowalski, Anna Golda, Michal Nowakowski, Agnieszka Skowron

**Affiliations:** 1 Department of Social Pharmacy, Faculty of Pharmacy, Jagiellonian University Medical College, Medyczna 9 Street, 30-688 Cracow, Poland; 2 Department of Medical Education, Jagiellonian University Medical College, Cracow, Poland

**Keywords:** OSPE, OSCE, PBL, Competence, Competence framework, Pharmacy students, Self-assessment

## Abstract

**Background:**

In addition to vocational education and experience, the pharmaceutical profession’s proper pursuit requires acquiring and continuously improving professional competencies. In recent years, the need has been increasingly highlighted for developing a medical education system based on helping students develop their competencies. It is necessary to adapt the tools and methods for assessing competencies during formal education. It will enable students to know the directions of further personal or professional development.

**Objective:**

The study aimed to compare pharmacy students’ self-assessment outcomes before and after the Objective Structured Practical Examination (OSPE), which finished the Pharmaceutical Care course (PCc). The study’s purpose was also to compare the outcomes of the self-assessment of competencies between the students of two academic years for whom classes on the PCc were provided by different methods.

**Methods:**

The study was conducted over two academic years (2018/2019 and 2019/2020) among 5th-year students enrolled at the Faculty of Pharmacy of the Jagiellonian University Medical College (JUMC) at the end of the Pharmaceutical Care course. Different teaching methods were used in the delivery of the course in these academic years. The students self-assessed their competencies using a questionnaire consisting of a list of personal and patient care competencies. The students completed the questionnaire before and after the OSPE, which followed the completion of the PCc.

**Results:**

Students’ professional competencies as self-assessed after the exam were higher than those assessed before the exam. Differences were observed in both personal and patient care competencies. Students taking the course in the 2019/2020 academic year set their pre-OSPE competencies higher than students taking the PCc in 2018/2019.

**Conclusion:**

The self-assessment scores increased for most competencies included in the study following the OSPE. This may suggest that taking part in the exam, involvement in patient’s case simulations, and self-assessment of performance at individual stages of the exam contributed to increased subjective assessment of professional competencies.

## Introduction

The role of a pharmacist evolves along with the changes in expectations placed upon the profession. In the healthcare system, the pharmacist’s role is no longer limited to distributing and ensuring the appropriate quality of medications. The pharmacist should supervise the patients’ use of medicines to ensure the safety and efficacy of pharmacotherapy [1]. Pharmaceutical care involves cooperation between the pharmacist and the patient to monitor the patient’s pharmacotherapy and consequently improve his/her quality of life [2]. According to the relevant European Directive, a pharmacist’s profession is a “regulated profession” and one of its main objectives: catering to the health of the patients [3].

In the EU, a pharmacist is a person who has completed a curriculum in university-level pharmaceutical education, including 6-month pharmacy internship, to become fully prepared for the unsupervised, full-responsibility provision of pharmaceutical services within a pharmacy setting [4]. One of these services consists of pharmaceutical care; as part of providing it, the pharmacist cooperates with the physician to monitor the patient’s pharmacotherapy to improve his/her quality of life [5]. In Poland, studies in pharmacy last 11 semesters, of which the last semester concerns the 6-month pharmacy internship.

Some of the most important responsibilities of the pharmacist include establishing professional contact with the patient and ensuring that the pharmacotherapy they receive is appropriate and safe [6]. The curricula established for pharmacy students are based on educational standards that identify the general and specific learning outcomes, methods for verifications of these outcomes being achieved in the educational process, and other requirements necessary for full vocational preparation [4]. The learning outcomes correspond to the 7th level of the Polish Qualifications Framework and assume an advanced level of pharmaceutical knowledge and professional skills being acquired by the students [7].

Competencies are defined as “being able to perform tasks and roles to the expected standard” [8]. In pharmacy, professional competence combines three attributes: knowledge, skills, attitude & experiences, and personal traits reflecting one’s capability to perform job-related tasks and functions consistent with the accepted legal standards of law and social expectations [9, 10]. One of the attributes of competencies is skills. Currently, more emphasis is put on students acquiring skills necessary to provide pharmaceutical services according to new educational standards; thereby, the teaching in pharmacy is more patient-centered [11].

Patient-centered care includes pharmacy professional skills and focuses on interpersonal relations and interacting effectively and harmoniously with patients. These are the so-called soft skills. Soft skills are one of the important elements that contribute to the professional development of a pharmacist, particularly concerning patient-centered care. They include the ability to build relationships with patients and team members, to understand, respect, and appreciate different and diverse competencies of other team members, or the ability to manage stress in conflicting and challenging situations [12].

Hard skills include the specific knowledge and abilities required that directly determine the quality of the professional tasks performed. For example, one of the most important hard skills a pharmacist should possess is calculating correct dose and prescription processing. In the pharmaceutical profession, the knowledge acquired in formal higher and postgraduate education and subsequent professional work is also very important [13].

The pharmaceutical profession’s proper pursuit involves continuous expansion and improvement rather than simply acquiring professional competencies. For several years, researchers and academics have become increasingly interested in assessing pharmacists’ and pharmacy students’ professional competence [14–16]. As part of the “Quality Assurance in European Pharmacy Education and Training” (PHAR-QA), a consortium made up of representatives of European pharmaceutical faculties defined a list of competencies for the professional practice of pharmacists within the EU market. This list is the mainstay of the European system to ensure the quality of pharmacists’ education and training [17, 18]. For our study, the above-mentioned list of competencies was used in the questionnaire, as translated into Polish with subsequent validation by back-translation.

## Purpose of the study

The primary aim of this study was to examine the influence of OSPE (compare pharmacy students’ self-assessment outcomes prior and after OSPE) on the self-assessment of students ‘professional competencies. The secondary aim was to examine the PCc teaching methodology (PBL, *Problem-based Learning)* influence on students’ professional competencies self-assessment.

## Materials and methods

### Study setting and participants

The study was conducted using a proprietary survey developed at the Department of Social Pharmacy of the Jagiellonian University Medical College. The study population consisted of the 5th-year students enrolled at the Jagiellonian University Medical College Faculty of Pharmacy and participated in the Pharmaceutical Care course in 2018/2019 and 2019/2020. In the academic year 2018/2019, the PCc was delivered using the direct instruction method and the case study method (individual work and case discussion, NO PBL PCc). In the academic year 2019/2020, the course was delivered using the Problem-Base Learning (PBL PCc) method and the case study method (individual work and case discussion). The PCc was completed with an objective structured practical exam (OSPE) in both cases. It was the first OSPE exam for these students. The exam consisted of 6 stations assessing the knowledge and skills that students should acquire during the course. All information related to the organization and course of the exam, including the description of the station, has been described in a separate publication by Dymek et al. [19].

A traditional PBL format was used for the first 5 meetings out of 10 of the PCc. Students worked in groups of 10–11 persons. They were assessed for their substantive participation in the discussion, responsibility for the group’s work, and relating to each other. The tutor watched and assessed the students. In special situations, the tutor could give tips to the group, but he was not allowed to provide solutions, answer questions and make decisions for the group [20–22]. Each group of students received 3 different descriptions of the situation (one after the other) containing problems to be jointly solved in the field of pharmaceutical care and clinical practice (developing a care plan, identifying and solving drug problems, educating the patient). In addition, as part of the course, students had 2 meetings on counseling in self-treatment and 2 meetings with Medicines Use Reviews (MURs), which were conducted using the case study method.

The survey was conducted using the Online Survey Tools. Invitations to participate in the survey were sent directly to potential subjects via the Moodle platform. Participation in the survey was voluntary and required the subject’s approval.

**Fig. 1:**
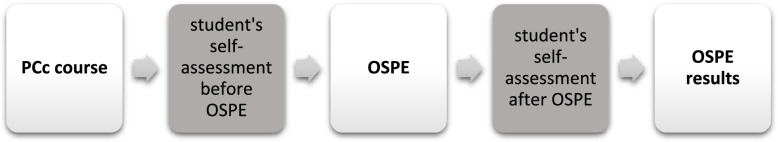
Figure design

The survey questions were provided 7 days before starting the OSPE session, and 7 days after all students had taken their exams, before the results’ announcement [19]. The survey was not available during the examination session (Fig. 1). Each student received a unique code they used for logging into the survey system on both occasions. At each stage of the study, the questionnaire could be completed only once by each student. Fully completed questionnaires from subjects participating in both parts of the survey were included in the analysis.

### The survey instrument development

The questionnaire was developed based on a list of competencies published by a consortium of European pharmaceutical department representatives within the PHAR-QA project [18]. The list of competencies was translated into Polish with subsequent validation by back-translation. The questionnaire list consisted of 24 personal competencies and 26 patient care competencies. Self-assessments of all 50 competencies were made by students using a 10-point scoring scale where a “1” score corresponds to an absolute lack of a particular competency, and “10” corresponds to a competency being mastered to perfection, with activities being performed by themselves while unsupervised, and fully responsible for their actions. Self-evaluation of competency is not a part of the curriculum so the survey was conducted for the purposes of the study.

Four researchers from the Department of Social Pharmacy of the JUMC assigned scores (0 to 3) to competencies depending on the ability to obtain competencies during the PCc. Further analysis included identifying and ranking competencies (assigned with a score of 1 or higher) by at least 2 out of 4 researchers. The average score for each competency was then calculated. A total of 29 competencies that would best acquire during the PCc (including 9 personal competencies and 20 patient care competencies) were selected for further analysis. Table 2 lists the 29 competencies chosen for the study in decreasing rank order.

### Data analysis

The data were analyzed using Statistica™ 12 package. This study used non-parametric tests. Wilcoxon signed-rank sum test was used to assess the results of self-assessments before and after the OSPE, and Mann-Whitney U-test was used to verify the statistical significance of differences between the results obtained in academic years 2018/2019 and 2019/2020. Statistical significance was set at *p* < 0.05.

## Results

The study group consisted of a total of 114 5th-year students enrolled at the Faculty of Pharmacy in two consecutive academic years, corresponding to 61.30% of the total number of 5th-year students, namely 63 students (57,80%) in the year 2018/2019 and 51 students (66,23%) in the year 2019/2020. All the enrolled students completed the survey twice. The average self-assessment of competencies was higher in students taking the survey in 2019/2020 than in 2018/2019 in both pre-and-post-OSPE surveys. In both academic years, post-OSPE self-assessments were higher than the corresponding pre-OSPE self-assessments (average of 6.87 vs. 6.26 and average of 7.44 vs. 6.83, respectively). The difference in student performance between pre-and-post-OSPE was Δ = 0.61. Table 1 shows the overall results of the pre-and post-exam surveys in both academic years.

**Table 1:** Descriptive statistics of points obtained from pre-and-post-OSPE perceived competence (scale 1–10)

	2018/2019 NO PBL PCc		2019/2020 PBL PCc	
	pre-OSPE	post-OSPE	pre-OSPE	post-OSPE
Mean	6,26	6,87	6,83	7,44
Min	4,51	5,67	5,12	6,30
Max	7,98	8,21	8,46	8,52
Median	6,05	6,75	6,62	7,30

### Pre-OSPE vs. post-OSPE perceived competence

The analysis revealed a post-OSPE increase in self-assessment scores for 8 out of 9 personal competencies. Statistically significant differences were observed for two competencies in the 2018/2019 academic year and six competencies in 2019/2020. Although no change was observed in the academic year 2018/2019 regarding the ability to communicate in the locally relevant language, a statistically insignificant reduction was observed in the 2019/2020 academic year.

Similarly, the patient care competencies analysis observed an increase in post-OSPE self-assessment scores compared to pre-OSPE scores. For 15 out of 20 competencies, this difference was statistically significant in both academic years. Table 2 presents the differences in pre-and-post-OSPE self-assessment scores obtained by students in academic years 2018/2019 and 2019/2020.

**Table 2:**
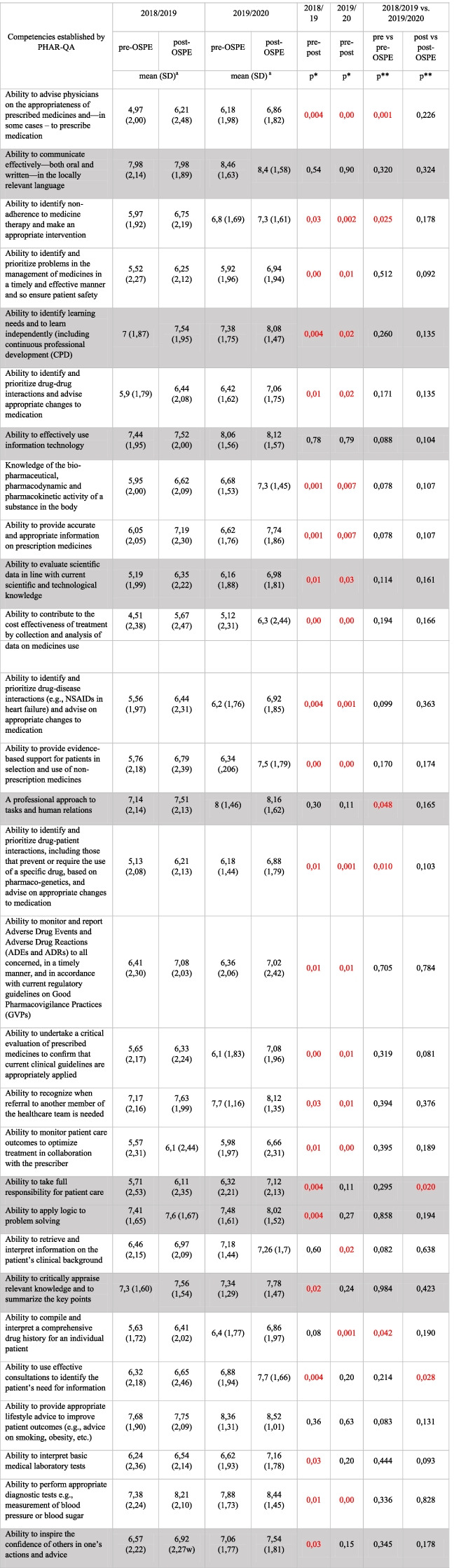
Student self-ratings of competence pre-and-post-OSPE (the order of competencies from the highest rank)

Rows of the table shown in gray– personal competencies

Rows of the table shown in white – patient care competencies

^a^Scale 1–10

*Evaluated using the Wilcoxon signed rank-sum test

**Evaluated using the Mann–Whitney U test

### Differences in pre-pre—OSPE and post-post-OSPE perceived competence for 2018/2019 and 2019/2020 academic year

Comparing pre-OSPE scores obtained by students in both academic years revealed that the reported scores were higher for all competencies in students taking the exam in the academic year 2019/2020 compared to those taking the exam the year before. For five competencies, the differences were statistically significant.

Competencies characterized by the most significant differences in self-assessment scores between individual academic years were related mainly to the ability to monitor and introduce changes in patient’s therapy, choice of medicines, detection of medication-related problems, including non-adherence to treatment drug-drug interactions.

## Discussion

The study was conducted among fifth-year pharmacy students who graduated from the PCc course and were admitted to the OSPE exam that ended this course. The pharmaceutical care course is carried out in the 9th semester of pharmaceutical studies. This course is comprehensively based on the knowledge and skills of students acquired in the previous years of study, among others in pharmacology, pathophysiology, pharmacotherapy, bromatology. The PCc is one of the last courses that students at the Faculty of Pharmacy pursue. This subject develops the ability to work with patients, among others conducting a pharmaceutical interview, identifying and solving drug problems, advising on self-treatment, educating the patient, developing a care plan based on the guidelines of scientific societies.

The OSPE exam, ending the PCc course assessing practical skills, is characterized by the same purpose and principles of designing and conducting as the OSCE exam, conducted among health professions students when assessing clinical skills [23]. Therefore, we compare the results of studies on OSPE and OSCE.

This study aimed to compare the outcomes of self-assessment of professional competencies of pharmacy students before and after the OSPE exam at the end of the PCc course and check the influence of OSPE and PBL on the self-assessment of students’ professional competencies.

Two variables were selected for the above study: OSPE and PBL, which may influence student self-assessment of competencies because they allow students to experience participation in situations that imitate the reality of a pharmacist’s work. PBL method simulates clinical practice and the situation from future professional life, students in small groups solve the problem from the pharmacy employees’ perspective providing the pharmaceutical service [24]. Moreover, during the OSPE, students participate in simulated situations that represent real events at work in a pharmacy (conducting a pharmaceutical interview, counseling on self-treatment, patient education) [23].

As part of the study, students assessed their professional competencies using a questionnaire prepared based on a list of competencies developed under the PHAR-QA project, “Quality Assurance in European Pharmacy Education and Training” [25]. Students assessed their competencies after completing the PCc before joining the OSPE exam, as well as for the second time after the exam. Before the exam, students assessed their professional competencies at an average level of over 60%. Out of 29 competencies assessed by students, there was an increase in self-assessment after OSPE in 28 cases. This increase was statistically significant for 23 competencies assessed by students of 2018/2019 and 18 of 2019/2020.

This result may indicate that the students defined their level of competence before the exam, evaluating some of them based on specific probabilities and assumptions. The exam allowed the students to verify their knowledge and skills. The form of conducting the exam, scenarios at individual stations, and contact with simulated patients made it possible to use the acquired knowledge and skills, which was reflected in an increase in the self-assessment of professional competencies. Completely different results were obtained by the team of Graves et al. researching, i.e., the relationship between student self-esteem (before and after OSCE) where self-assessment of competencies perceived by students decreased after participating in OSCE exam. The authors explained this phenomenon as the inability to predict and determine the level of one’s competencies before the exam, and OSCE itself did not change it [26].

In the published studies, the authors most often used the OSCE as a tool for assessing students’ competencies and for assessing the course itself [27–29]. Some authors also compare the exam results obtained by students during the assessment conducted by the examiner or the feelings of the simulated patient with the competencies perceived by students [30, 31]. According to the trends in teaching in medical and pharmaceutical sciences, as part of such a comprehensive course as Pharmaceutical Care, we give up teaching methods in favor of activating methods [32–34].

In the 2019/2020 academic year, classes were conducted using the Problem-based Learning (PBL PCc) method, which motivates students to acquire knowledge and individually search for possible solutions independently [32].

Therefore, another objective of the study was to compare the results of the self-assessment of professional competencies of pharmacy students of 2 years for whom the PCc classes were conducted using different methods. The results show that students from 2019/2020, in which the PBL method was introduced, rated their competencies higher even before OSPE compared to students from 2018/2019 (pre-OSPE = 6.26 vs. pre-OSPE = 6.83) with the direct instruction method.

Out of 29 assessed competencies, 28 students from 2019/2020 rated higher than students from 2018/2019. In 5 cases, this difference was statistically significant. These competencies relate to the patient’s pharmacotherapy, communication with the team, and contacts between professionals. This result is consistent with the results of other research in the field of didactics in the areas of medical and pharmaceutical sciences, where it is clear that practical skills, the use of knowledge, and coping in a team are acquired to a higher degree during activating methods compared to Conventional Teaching and Learning (CTL). This dependence is confirmed both in self-assessment tests and in examinations [35–37].

## Conclusion

The research results suggest that taking part in OSPE, meeting with simulated patients, and analyzing medication-related problems of simulated patients contributed to the increased subjective assessment of professional competence. Thus, OSPE is a form of learning for students.

Simultaneously, the results suggest that a change in the teaching method from passive to activating teaching methods (PBL) contributed to increased subjective self-assessment of professional competence before the OSPE. Therefore, the study results will contribute to subsequent changes in teaching methods to focus on students acquiring better professional competence.

## Data Availability

The datasets generated during and analyzed during the current study are not publicly available due to the Local Ethics board requiring these to be held securely by the research team members. Still, aggregate data are available from the corresponding author on reasonable request.

## Abbreviations

OSPE: Objective Structured Practical Examination
PCc: Pharmaceutical Care course
JUMC: Jagiellonian University Medical College
